# Health-related messages in the labeling of processed meat products: a market evaluation

**DOI:** 10.29219/fnr.v63.3358

**Published:** 2019-05-02

**Authors:** Diana Ansorena, Sandivel Cama, Marta Alejandre, Iciar Astiasarán

**Affiliations:** Department of Nutrition, Food Science and Physiology, Faculty of Pharmacy and Nutrition, Universidad de Navarra, IDISNA – Instituto de Investigación Sanitaria de Navarra, Pamplona, Spain

**Keywords:** nutrition claims, health claims, allergens, additives, packaged foods

## Abstract

**Background:**

Food labeling is an important communication tool for the exposure of nutrition information in foods.

**Objective:**

The presence of labeling messages related to nutrients, health properties, allergens, and additives in meat products marketed in Spain was analyzed in this work. The data collection was done through the web pages of six Spanish meat industries, and 642 products were gathered. The following labeling information was collected: the presence of nutrition claims, the presence of health claims, messages indicating the absence of additives, and those reporting the absence or presence of allergenic substances.

**Results:**

A total of 1,254 messages were found with the following distribution: 72% were related to the presence/absence of allergens, 19% were nutrition claims, 8% were messages related to the absence of additives, and only 0.4% were health claims. Fat was the nutrient most frequently referred in the nutrition claims, accounting for a 63.5% of this type of claims, with the expression ‘low-fat’, as the most used (42% of total nutrition claims). Prevalence of processed meat products that showed nutrition claims was 29%, whereas the percentage of products that showed information about allergenic compounds was 83%.

**Conclusion:**

This work provides information about the presence of health-related messages in a high number of meat products, which could be useful as a tool for marketing purposes or for consumer trends evaluation studies.

## Popular scientific summary

- Assessment of the labeling of 642 processed meat products reported 1,254 messages
- One hundred eighty-three products showed nutrition claims (29% of total), 5 products showed health claims (0.8% of total), whereas 536 products showed information about the presence/absence of allergens (83% of total)
- About 63.5% of the nutrition claims referred to the fat content or to some particular characteristics of the lipid profile.
- Labeling data reported in this work can be useful as tool for consumer market research

The information displayed on the food labeling can be a very important element in the purchasing decision of consumers (1), and it is also part of the marketing strategies of the food industry.

Today, food label information about different aspects related with health has special interest for consumers, especially to those who are highly health-oriented (2). One of these aspects is the information linked to the presence of allergens, which constitute toxic agents for some sensitive groups of consumers, so their presence must be declared in the labels, according to Regulation 1169/2011 (3). On the contrary, the ‘clean label’ trend has contributed to increase the information in foods not only about the absence of allergenic products, but also about the absence of additives (4). As they have to be used only in case they are needed, their absence is sometimes related with a better quality of raw materials or to better processing technologies. In fact, the food industry has started to respond to the increasing consumer demand of such clean label products by supplying foods that are perceived as ‘cleaner’ (5).

Also, nutrition and health claims are nowadays more frequently present in the food labels, answering to the demand of the consumers about information useful to contribute to prevent some diseases and to obtain healthy benefits. Moreover, it has been reported that while consumers interested in household well-being are particularly focused on nutrition claims, health claims seem to be of interest for the more vulnerable segments of population (6).

The use of different types of claims is regulated in many countries to ensure a high level of protection for consumers, to facilitate their choices, and to avoid misleading information, among other reasons. In the European Union, the Regulation 1924/2006 establishes the definitions of nutrition and health claims, the conditions to be applied in each case, and the composition criteria to be fulfilled by the different types of nutrition claims (7). Thus, a nutrition claim is any statement that states, suggests, or implies that a food has beneficial nutritional properties because of the energy it provides – provides at a reduced or increased level or does not provide – and/or nutrients or other substances it contains – contains in reduced or increased ratios or does not contain. On the contrary, a health claim is any statement that affirms, suggests, or implies that there is a relationship between a category of food, a food or one of its components, and health. Moreover, the Regulation 432/2012 sets the conditions needed to use some of the general function health claims (8).

It has been noticed that the implementation of these norms varies across countries and also between different food categories (9, 10). Hieke et al. pointed out that while 30% of foods sampled in the United Kingdom carried a nutrition claim, only 16% of products showed this type of information in Germany (10). Moreover, it has been described that consumer evaluations of different types of nutritional and health claims can also vary across countries and depend on whether or not they have some prior knowledge concerning added healthy ingredients (11). For instance, in a study that evaluated consumer preferences for beef with nutrition and health claims, it was observed that in Belgium, the Netherlands, and France, nutrition and health claims on saturated fat were more attractive to consumers than claims on protein and/or iron, whereas the opposite was found among consumers in the United Kingdom (12).

On the contrary, the interest in maintaining a healthy diet has led to a significant development of functional foods in which it is also sought to show its benefits through labeling. In this context, it has been reported that the type of claim, consumer group, carrier, and claim wording play an important role in consumer perception of functional foods, which result in different functional efficacy expectations and (re)purchase intent (13).

In the particular case of the meat industry, as occurs in other food sectors, extensive research has been done in the development of functional products. It is undergoing major changes as a result of continuous technological innovations and changes in consumer demands, including those related to the search for a healthier diet (14, 15). It has been pointed out that nutrition claims may help to provide the consumer with a means of trusted information in relation to the nutrients content of meat, and the industry sector with tools to highlight particular nutritional properties of meat (16). In this sense, it is worth noting that meat products have been classified as good carriers for functional foods (1, 17).

The objective of this work was to analyze the presence of health-related messages that refer to nutrients, health properties, allergens, and additives in meat products currently marketed in Spain. The results obtained will contribute to provide useful information for marketing purposes and consumer trends evaluation studies.

## Materials and methods

Labeling data were collected from prepackaged meat products, excluding unpackaged fresh meat, and they were obtained through the websites of six Spanish meat products companies. These companies were selected according to production and marketing volume criteria. In total, they account for approximately 80% of the Spanish meat market share (18).

‘Meat products’ are defined as processed products resulting from the processing of meat or from the further processing of such processed products, so that the cut surface shows that the product no longer has the characteristics of fresh meat (19). Classification of the sampled products (*n* = 642) was done following criteria of the currently in-force norm for meat products in Spain (20). This norm classifies meat products in two major groups: heated meat products (including the following subgroups: sterilized, pasteurized, and treated by incomplete heat-treatment products) and non-heated meat products (including the following subgroups: dry-cured, airy, marinated, brined, and non-treated products). Within each subgroup, different types of products were found, accounting for a total number of 77 types of products (Supplementary Table 1).

For each of the 642 products, the following labeling information was collected into a Microsoft Excel spreadsheet: the presence of nutrition claims, the presence of health claims, messages indicating the absence of additives, and those reporting the absence or presence of allergenic substances. Definitions of the EU Regulation 1924/2006 for both nutrition and health claims were considered for that purpose (7). Moreover, nutrition claims were also classified depending on the type of nutrient that was mentioned in the claim (sugar, protein, fat, type of fat, vitamins, or minerals). Information about substances causing allergies or intolerances was gathered if they were mentioned in the list shown in the Annex II of EU Regulation 1169/2011 (3). The number and type of claim in every product was collected.

## Results and discussion

### General information about analyzed meat products

Labels of 642 different meat products were examined, and information about nutrition claims, health claims, and about the absence of additives or substances causing allergies or intolerances was gathered and analyzed in this work. These 642 products were classified into two major categories: heat-treated meat products (*n* = 373) and non-heat-treated meat products (*n* = 269) (Table 1). Among the heat-treated products, the largest proportion corresponded to the pasteurized products (*n* = 365), which accounted for 57% of total products. Among non-heat-treated products, dry-cured subgroup showed the highest number of meat products (*n* = 194), accounting for 30% of total products. Therefore, approximately 90% of the meat products marketed in Spain corresponded to two subgroups: pasteurized and dry-cured products. In particular, the types of meat products with the greatest presence in this study were cooked ham (*n* = 56) and turkey breast (*n* = 51) among the heat-treated products, and chorizo (*n* = 38) among the non-heated ones (Supplementary Table 1).

**Table 1:** Number and percentage of meat products analyzed in each subgroup of meat product

Category of meat products	Total number of meat products	Total meat products (%)
Heat-treated meat derivatives	373	58
Sterilized	3	0
Pasteurized	365	57
Incomplete heat treatment	5	1
Non-heat-treated meat products	269	42
Dry-cured	194	30
Airy	0	0
Marinated	30	5
Brined	0	0
Not treated	45	7
Total	642	100

### Analysis of meat products with or without messages (nutrition, health properties, allergens, and additives)

After this general overview of the number and type of meat products marketed in Spain, the different types of messages shown on their labels were classified and quantified.

Among the 642 meat products examined, there were 183 products with nutrition claims, 5 products with health claims, 74 products with statements about the absence of additives, and 536 products with statements related to the presence or absence of allergens (Table 2). These data mean that about 29% of total products included nutrition claims, 12% provided information about the presence of additives, and only 0.8% included health claims. It should be noted that 83% of the products presented information on the presence or absence of allergens in their labeling, being the expressions ‘lactose-free’ or ‘soy-free’ the most frequently found.

**Table 2:** Number of meat products with different health-related messages in each category of meat product

Category of meat products	Products with nutrition claims	Products with health claims	Products with information about	
			Additives	Allergens
Heat-treated meat derivatives				
Sterilized	0	0	0	2
Pasteurized	128	0	56	324
Incomplete heat treatment	4	0	0	4
Non-heat-treated meat products				
Dry-cured	15	5	18	138
Airy	0	0	0	0
Marinated	17	0	0	26
Brined	0	0	0	0
Not treated	19	0	0	42
Total	183	5	74	536

Among all the products analyzed, only 11 meat products (2% of total) did not show any kind of the assessed messages, among which ham cream (*n* = 3) and Iberian loin (*n* = 2) were the most common.

### Analysis of the types of messages present in the labeling of meat products

Each product usually provided varied information, showing, in most cases, more than one type of message. Thus, the total number of messages in each product was counted and classified.

A total of 1,254 messages were found in the 642 meat products analyzed. They were distributed in nutrition claims (*n* = 236), health claims (*n* = 5), additives-related messages (*n* = 105), and allergens-related messages (*n* = 908). As pasteurized products were the major group in terms of number of products found, it also accounted for the greatest number of total messages (69%), as expected. In contrast, the sterilized ones were those with the lowest presence of different health-related messages (only 0.16% of the total).

**Fig. 1:**
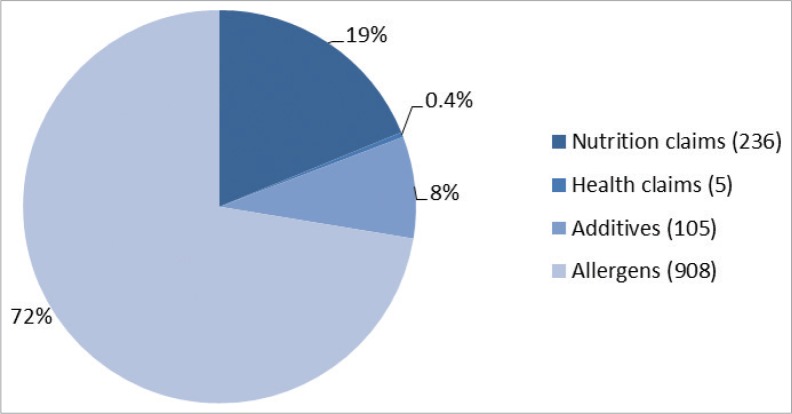
Distribution total number of health-related messages (*n* = 1,254) present in the meat products. Between parentheses, the number of messages reported for each type.

As can be seen in Fig. 1, these data meant that 73% of the total number of messages were related to the presence or absence of allergens, followed by the nutrition claims with 19%, then with a lower proportion of claims linked to the absence of additives and health claims with 8 and 0.4%, respectively.

Another fact to mention is that there were products with more than one nutrition claim on their label. Actually, the product with the greatest number of nutrition claims on a single product was the ‘sausage with cheese’ that included three claims (‘source of protein’, ‘source of phosphorous’, and ‘low sugar content’). Other 13 products showed two claims each, and the rest, only one.

Finally, only five health claims were found on the labels of the 642 meat products. In particular, the statement found was ‘feed your defenses’ that was present in the following dry-fermented products: chorizo, turkey-chorizo, salami, salami, and turkey-sausage. This statement was associated, in these products, with the presence of relevant amounts of vitamin B12 and iron. In fact, the EU Regulation 432/2012 authorizes the health claim ‘contributes to the normal function of the immune system’ for foods that are at least ‘source of [vitamin B12 or iron]’, namely, that 100 g supply 15% of the daily reference intake for these two nutrients, according to Annex XIII of EU Regulation 1169/2011 (3, 8). As it can be observed, the original wording of the claim has been slightly modified, maintaining the same meaning, as it is allowed by Recital 9 of the Regulation 432/2012 (8).

### Analysis of nutrition claims

A more detailed evaluation of the type of nutrition claims found in this work was done. The nutrients referred in the nutrition statements on the labels of meat products were sugar, protein, fat, some minerals and vitamin B12 (Table 3). More than half of the nutrition claims (63.5%) referred to the fat content or to some particular characteristics of the lipid profile, whereas approximately one-fifth (22.4%) referred to the presence of certain micronutrients. The two protein-related nutrition claims accounted for a 13.5% of total nutrition claims.

**Table 3:** Types of nutrition claims referred in the analyzed meat products

Nutrient	Nutrition claims	Number of claims in meat products	Total nutrition claims (%)	
Sugar	Low sugars	1	0.4	Sugar = 0.4
Protein	Source of protein	9	3.8	Protein = 13.5
	High protein	23	9.7	
Fat	High monounsaturated fat	4	1.7	
	Low fat	98	41.5	Fat = 63.5
	Low saturated fat	3	1.3	
	Reduced cholesterol	7	3.0	
	Reduced fat	18	7.6	
	Fat free	20	8.5	
Vitamins and Minerals	Reduced sodium/salt	37	15.7	
	Low sodium/salt	1	0.4	Vitamins and minerals = 22.4
	Source of phosphorus	5	2.1	
	Source of iron	5	2.1	
	Source of vitamin B12	5	2.1	
	Total	236		

In particular, the statement ‘low-fat’ was, with great difference, the most prevalent nutrition claim found in meat products, accounting for a 41.5% of the total amount of nutrition claims. It was followed by the claim ‘reduced sodium/salt’ (15.7%). These two claims are consequence of the implementation of reformulation strategies that are currently leading to achieve healthier products (21). In this sense, EFSA proposed that the intake of saturated fatty acids should be as low as possible (22), whereas the European Union oversees the salt reduction initiatives intended to reach the recommended intake levels (23). In particular, the Health Canada’s food guide recommends that if processed meats are consumed, consumers should choose lower fat and sodium varieties (24). Moreover, according to Shan et al. (25), processed meats with reduced salt and/or fat positively influence purchase intention and health perception. It has also been pointed out that consumers attribute a great importance to vitamins and minerals, together with omega-3 fatty acids, when recognizing the health benefits of functional foods (1).

Nutrition claims such as ‘high protein’ and ‘fat free’ had also a relevant presence on the labels of meat products, with 9.7 and 8.5%, respectively, of the total number of nutrition claims.

### Comparison with other studies

The type of analysis carried out in this study has few precedents in the literature, so it is difficult to assess or compare this information with other studies in which either the methodology or the sampling conditions were different. It is generally recognized that there is a worldwide interest in assessing whether the information on food labeling is related to the image of the food industry, to detect if there are food sectors where the inclusion of claims is more frequent, or to assess whether there are differences between countries when applying existing legislation.

In this sense, the prevalence of nutrition and health claims has been an issue of interest in countries where their regulations are already well established, such as the United States, Canada, Australia, and New Zealand, but studies on the European market are still scarce (10), in particular those related to meat products. Table 4 summarizes current data of studies that have particularly evaluated the presence of nutrition and health claims in processed meat. No data about presence or absence of additives has been found in the literature for comparison purposes. In relation to allergenic compounds, labeling data have been reported in different food sectors, one of them being ‘delicatessen meat’, but no information on processed meat products is shown (26).

**Table 4:** Prevalence of nutrition claims in meat products commercialized in some European countries

Country (year)	Total number of meat products	Products with nutrition claims (%)	Reference
Ireland (2009)	64	16	(27)
Serbia (2014)	452	8	(29)
UK^a^ (2015)	105	16.2	(28)
Slovenia (2016)	429	15(c); 8(d)	(9)
UK, Spain, Slovenia, the Netherlands, Germany^b^ (2016)	189	9.5	(10)
Spain (present work)	642	29	-

a In this study, meat products were included in the same group as fish and processed meals, so the real number of meat products would be lower.

b Data in this study include results from all the five countries.

c This data represents the percentage of available food products with claims.

d This data represents the percentage of sold food products with claims.

In Ireland, nutrition and health claims were studied on the most heavily consumed packaged products on the Irish market. Processed meat category showed that among 64 products examined, 10 showed at least one nutrition claim (16%), the most of which referred to ‘fat’, as it has also been observed in our work. However, these authors did not find health claims in the analyzed meat products (27).

Another study in the United Kingdom examined the labeling of several food categories from the home-shopping website of a well-known retailer. One of the categories of food merged fish products, meats, and prepared meals, with a total number of 105 products. In this category, 17 products with nutrition claims and 10 products with health claims were found (28).

The exposure of consumers to nutrition and health claims in different categories of prepackaged foods was also assessed in Slovenia (9). They observed that among the 429 meat products evaluated, 15% of those available on stores and 8% of purchased products included nutrition claims, being again fat the most referred nutrient. This study reported the presence of health claims in this type of foods (up to 7% of products), a higher rate as compared to our data (0.4%). However, in the case of Serbia, 452 meat products were assessed, showing an 8% of nutrition claims and 0.1% of health claims.

Within the context of the CLYMBOL project (10), a study of five European countries reported the current status of nutrition and health claims, analyzing 2,034 foods and drinks. The United Kingdom was the country with the highest prevalence of nutrition claims (29.6%), followed by Spain (23%), Slovenia (18.8%), the Netherlands (16.8%), and Germany (16%). Health claims showed slightly lower variation in the five countries, being highest in Slovenia and Germany (both 37%), followed by the Netherlands (31%), Spain (24%), and the United Kingdom (21%). One of the categories of foods analyzed was meat and meat products, where 184 products were analyzed in total. Eighteen of them presented nutrition claims (9.5% of the total of products), and 13 showed health claims (5.8%). No detailed information for meat products was reported for every country in that work.

As it can be seen, prevalence of nutrition claims is significantly higher in our study (29%) as compared to the prevalence of this type of claims reported in previous papers for this food category. Different reasons could be hypothesized for this finding: marketing strategies could be differently used by companies, and implementation of the EU regulation might vary across countries, as stated by other authors.

## Conclusion

In summary, this work provides information about the presence of health-related messages in a high number of Spanish meat products, which could be useful as a tool for marketing purposes or for consumer trends evaluation studies. It is worthy to highlight that 1,254 health-related messages were gathered from the 642 analyzed products, of which 183 showed nutrition claims (29% of total), 5 products showed health claims (0.8% of total), and 536 products showed information about the presence/absence of allergens (83% of total). In addition, ‘low-fat’ was the most used nutrition claim.

## Supplementary Material

Health-related messages in the labeling of processed meat products: a market evaluation
