# Supplementary material for: Health-related messages in the labeling of processed meat products: a market evaluation
Source: Food Nutr Res. 2019 May 2;63:10.29219/fnr.v63.3358. doi: 10.29219/fnr.v63.3358 (PMC6503640; doi:10.29219/fnr.v63.3358)
Supplement: Health-related messages in the labeling of processed meat products: a market evaluation [file FNR-63-3358-s001.docx]

Table S1. Number of products analysed per type of meat product and company

|  |  | **Types of meat products** | **Companies** | | | | | | **Number of products** |
| --- | --- | --- | --- | --- | --- | --- | --- | --- | --- |
|  |  |  | **C1** | **C2** | **C3** | **C4** | **C5** | **C6** |  |
| ***Heat treated products*** | | |  |  |  |  |  |  |  |
| **Sterilized** | |  |  |  |  |  |  |  | **3** |
|  | 1 | Canned chopped |  | 1 |  |  |  | 1 | 2 |
|  | 2 | Canned turkey ham |  |  |  |  |  | 1 | 1 |
| **Pasteurized** | |  |  |  |  |  |  |  | **365** |
|  | 3 | Butifarra |  |  |  |  | 1 |  | 1 |
|  | 4 | Chopped | 4 | 8 |  |  |  | 1 | 13 |
|  | 5 | Beef chopped | 3 |  |  |  |  |  | 3 |
|  | 6 | Turkey chopped | 4 | 5 | 2 |  |  | 2 | 13 |
|  | 7 | Cooked ham cream |  | 3 |  |  |  |  | 3 |
|  | 8 | Turkey cream |  | 1 |  |  |  |  | 1 |
|  | 9 | Cooked ham cold cut |  | 13 |  |  |  |  | 13 |
|  | 10 | Lean meat cold cut |  |  |  | 1 | 1 |  | 2 |
|  | 11 | Turkey breast cold cut | 10 | 11 |  | 3 | 3 | 5 | 32 |
|  | 12 | Chicken breast cold cut | 6 | 2 |  |  | 3 | 3 | 14 |
|  | 13 | York ham cold cut | 1 | 8 | 2 | 2 |  | 7 | 20 |
|  | 14 | Cooked front leg cold cut | 2 | 2 |  |  | 1 |  | 5 |
|  | 15 | Galantines |  | 1 |  |  | 1 |  | 2 |
|  | 16 | Veggie galantines |  |  |  |  | 1 |  | 1 |
|  | 17 | Cooked ham | 9 | 13 | 13 | 6 | 8 | 7 | 56 |
|  | 18 | Turkey cooked ham | 2 | 4 |  |  | 1 | 2 | 9 |
|  | 19 | Lacon (sliced) |  |  |  |  |  | 1 | 1 |
|  | 20 | Mortadella | 7 | 16 |  | 4 | 4 | 4 | 35 |
|  | 21 | Turkey mortadella | 3 | 8 |  | 1 |  |  | 12 |
|  | 22 | Veggie mortadella |  |  |  |  | 1 |  | 1 |
|  | 23 | Cooked front leg |  |  | 1 |  |  | 5 | 6 |
|  | 24 | Turkey breast | 6 | 16 | 15 | 5 | 5 | 4 | 51 |
|  | 25 | Chicken breast | 4 | 5 | 3 |  | 2 | 1 | 15 |
|  | 26 | Cooked sausages | 10 | 6 | 4 |  |  | 9 | 29 |
|  | 27 | Turkey cooked sausages | 4 | 2 | 1 |  |  | 1 | 8 |
|  | 28 | Chicken cooked sausages | 5 |  |  |  |  |  | 5 |
|  | 29 | Cooked ham sausages | 1 |  |  |  |  |  | 1 |
|  | 30 | Sausages with milk | 1 | 2 |  |  |  |  | 3 |
|  | 31 | Sausages with cheese |  | 2 | 2 |  |  |  | 4 |
|  | 32 | Sobrasada |  | 6 |  |  |  |  | 6 |
| **Incomplete heat treatment** | |  |  |  |  |  |  |  | **5** |
|  | 33 | Turkey bacon |  | 1 |  |  |  |  | 1 |
|  | 34 | Turkey bacon cold cut | 2 |  | 1 |  |  |  | 3 |
|  | 35 | Chicken bacon cold cut | 1 |  |  |  |  |  | 1 |
| ***Non heated products*** | | |  |  |  |  |  |  |  |
| **Dry-cured** | |  |  |  |  |  |  |  | **194** |
|  | 36 | Smoked bacon | 2 | 4 |  | 2 |  | 9 | 17 |
|  | 37 | Chistorra |  |  | 1 |  |  |  | 1 |
|  | 38 | Chorizo | 1 | 4 | 4 | 13 | 4 | 12 | 38 |
|  | 39 | Turkey chorizo |  | 2 |  |  |  |  | 2 |
|  | 40 | Iberian chorizo |  | 1 | 1 |  |  | 1 | 3 |
|  | 41 | Fuet | 1 | 11 | 1 | 12 | 8 | 1 | 34 |
|  | 42 | Turkey fuet | 1 | 2 |  |  |  |  | 3 |
|  | 43 | Cured ham |  | 8 | 3 | 4 | 5 | 3 | 23 |
|  | 44 | Turkey cured ham |  |  | 2 |  |  |  | 2 |
|  | 45 | Iberian ham |  | 1 |  |  |  | 1 | 2 |
|  | 46 | Smoked lacon |  |  |  |  |  | 1 | 1 |
|  | 47 | Dried loin |  |  | 2 | 1 | 1 | 11 | 15 |
|  | 48 | Iberian loin |  | 1 | 1 |  |  |  | 2 |
|  | 49 | Longaniza |  |  | 1 | 1 |  | 1 | 3 |
|  | 50 | Blood iberian sausage |  |  |  |  |  | 1 | 1 |
|  | 51 | Iberian front leg |  | 3 |  |  |  | 1 | 4 |
|  | 52 | Pepperoni |  |  |  |  |  | 3 | 3 |
|  | 53 | Salami | 1 | 3 |  | 2 | 2 | 4 | 12 |
|  | 54 | Fermented sausage (Salchichón) | 1 | 5 | 1 | 4 | 6 | 7 | 24 |
|  | 55 | Turkey fermented sausage |  | 1 |  |  |  |  | 1 |
|  | 56 | Iberian fermented sausage |  | 1 | 1 |  |  | 1 | 3 |
| **Airy** | |  |  |  |  |  |  |  | **0** |
| **Marinated** | |  |  |  |  |  |  |  | **30** |
|  | 57 | Marinated ham |  | 2 |  |  |  |  | 2 |
|  | 58 | Lacón a la gallega |  |  |  |  |  | 1 | 1 |
|  | 59 | Marinated loin |  | 5 |  |  |  | 5 | 10 |
|  | 60 | Marinated turkey loin |  | 1 |  |  |  |  | 1 |
|  | 61 | Sajonia loin |  | 3 |  |  |  |  | 3 |
|  | 62 | Marinated-garlic lean meat |  | 4 |  |  |  | 1 | 5 |
|  | 63 | Marinated lean meat |  | 2 |  |  |  |  | 2 |
|  | 64 | Marinated breast turkey |  | 6 |  |  |  |  | 6 |
| **Brined** | |  |  |  |  |  |  |  | **0** |
| **Not treated** | |  |  |  |  |  |  |  | **45** |
|  | 65 | Turkey and pork meatballs |  | 2 |  |  | 1 |  | 3 |
|  | 66 | Butifarra |  |  |  |  | 4 |  | 4 |
|  | 67 | Beef burguer |  | 4 |  |  | 1 |  | 5 |
|  | 68 | Poultry burguer |  | 7 |  |  |  |  | 7 |
|  | 69 | Pork burguer |  | 5 |  |  |  |  | 5 |
|  | 70 | Beef and pork burguer |  | 2 |  |  | 3 |  | 5 |
|  | 71 | Longaniza |  | 2 |  |  |  |  | 2 |
|  | 72 | Longaniza (turkey) |  | 1 |  |  |  |  | 1 |
|  | 73 | Ground poultry meat |  | 2 |  |  |  |  | 2 |
|  | 74 | Ground pork meat |  | 2 |  |  | 1 |  | 3 |
|  | 75 | Ground pork and beef meat |  | 3 |  |  | 1 |  | 4 |
|  | 76 | Ground beef meat |  | 1 |  |  | 1 |  | 2 |
|  | 77 | Fresh sausage |  |  |  |  | 2 |  | 2 |
| **Total** | |  | **92** | **237** | **62** | **61** | **72** | **118** | **642** |
